# Factors associated with a double burden of malnutrition among preschool-aged ethnic minority children in northern Thailand: a community-based cross-sectional study

**DOI:** 10.1186/s12889-026-26777-8

**Published:** 2026-02-25

**Authors:** Siwarak Kitchanapaibul, Chomnard Singhan, Thapakorn Ruanjai

**Affiliations:** 1 School of Health Science, Mae Fah Luang University, Chiang Rai, 57100 Thailand; 2 School of Medical Science, University of Phayao, Phayao, 56000 Thailand

**Keywords:** Double Burden of Malnutrition, Preschool-Aged Children, Ethnic Minority Group, Northern Thailand

## Abstract

**Background:**

The double burden of malnutrition (undernutrition and overnutrition) remains a major public health concern among preschool-aged ethnic minority children in northern Thailand. Cultural and geographic contexts shape caregiving and dietary behaviors while limiting access to health and nutritional services.

**Objective:**

This study examined the relationship between the double burden of malnutrition and its associated behavioral and socioenvironmental factors among preschool-aged ethnic minority children.

**Methods:**

A community-based cross-sectional study was conducted among 260 children aged 2–5 years in Chiang Rai Province, northern Thailand. Data on sociodemographic, caregiving, and dietary characteristics were obtained from parents or caregivers using structured questionnaires, and the nutritional status of the children was assessed via WHO-standardized anthropometric measurements. Multivariate logistic regression was used to identify factors associated with stunting, wasting, and overweight/obesity.

**Results:**

The findings revealed a high prevalence of the double burden of malnutrition, including stunting (22.3%), wasting (20.8%), and overweight/obesity (11.9%). Stunting was associated with frequent consumption of staple starchy foods (OR 9.03; 95% CI 1.18–69.05). Wasting was associated with meals prepared by nonparental caregivers (OR 2.51; 95% CI 1.12–5.64). Overweight/obesity was related to mothers being the primary income earners (OR 3.14; 95% CI 1.23–8.04) and having low vegetable intake (OR 2.35; 95% CI 1.07–5.20).

**Conclusion:**

The coexistence of undernutrition and overnutrition among ethnic minority preschoolers is driven by both behavioral and socioenvironmental determinants. Targeted interventions that enhance caregiver nutrition literacy, promote healthy feeding behaviors, and integrate qualified nutrition professionals into community-based childcare systems are crucial for addressing these nutritional disparities.

**Supplementary Information:**

The online version contains supplementary material available at 10.1186/s12889-026-26777-8.

## Introduction

Proper nutrition is crucial for early childhood growth and development, as it provides essential components that the body needs for physical growth, the nervous system, brain function, and the enhancement of intellectual ability and intelligence. In addition, meeting nutritional requirements is essential for achieving growth and developmental outcomes that align with a child’s genetic potential [1]. Healthy growth in children can be observed through measurable changes in body size and structure. Anthropometric assessments serve as key indicators of growth [2], which facilitating the early detection and monitoring of child malnutrition. The coexistence of undernutrition and overnutrition within the same population, household, or individual is referred to as the double burden of malnutrition (DBM). DBM has emerged as an increasing public health challenge in low- and middle-income countries (LMICs), particularly among children [3–5]. This condition contributes substantially to child morbidity, mortality, and long-term developmental deficits.

In 2022, an estimated 148.1 million children under five years of age globally (22.0%) were stunted (low height-for-age, H/A), 45 million (6.8%) were wasted (low weight-for-height, W/H), and 37 million (5.6%) were overweight (high weight-for-height, W/H) [6]. In terms of regional patterns, Asia accounted for the largest burden of childhood malnutrition, with 53% of all children affected by stunting, 70% of those affected by wasting, and 48% of those affected by overweight residing in the region [6]. Southern Asia has the highest prevalence of both stunting and wasting, followed by Southeast Asia [6, 7]. In Thailand, a Southeast Asian country, the prevalence rates among children under five years of age in 2022 were 11.8% for stunting (H/A ≤ − 2 SD), 7.7% for wasting (W/H ≤ − 2 SD), and 8.6% for overweight and obesity (W/H ≥ + 2 SD) [6, 8]. Moreover, among Thai preschool children aged 3–5 years in the same year, the prevalence rates of stunting, overweight/obesity, and wasting were 10.8%, 9.9%, and 5.9%, respectively [9].

Numerous studies have examined factors associated with the occurrence of the double burden of malnutrition. These studies have identified a range of contributing factors, including early-life nutrition, sociodemographic and socioeconomic characteristics, behavioral factors, dietary diversity, food security, and educational status [10–15]. In LMICs, malnutrition among children under five years of age, including preschool-aged children, is often driven by a combination of inappropriate feeding practices, nutrient deficiencies, recurrent infections, and environmental and socioeconomic conditions [7, 12, 16–18]. These conditions are frequently exacerbated in rural and ethnically diverse communities, where geographic isolation, limited access to healthcare services, food insecurity, and cultural dietary practices create substantial barriers to optimal child growth and development [19, 20]. The double burden of malnutrition (undernutrition and overnutrition) remains a major public health issue among preschool-aged ethnic minority children in northern Thailand. In particular, Chiang Rai Province is home to many ethnic minority groups that experience significant health disparities [21]. These populations often face limited access to services and heightened vulnerability to malnutrition due to poverty, low levels of maternal education, inadequate health infrastructure, and social exclusion [22, 23]. These challenges are not unique to Thailand but are also observed in rural and ethnic minority populations in other LMICs, where structural inequities, cultural dietary norms, and limited healthcare access interact to shape child nutrition outcomes. However, few studies have examined the coexistence of undernutrition and overnutrition among ethnic minority preschool children in rural northern Thailand, and context-specific factors associated with malnutrition remain poorly understood. Understanding the double burden of malnutrition among preschool children in these communities, together with associated individual, behavioral, and environmental factors, can inform culturally tailored nutrition interventions and guide future policies aimed at promoting child health equity in northern Thailand. Therefore, this study aimed to examine factors associated with malnutrition, including stunting, wasting, and overweight, among preschool-aged ethnic minority children in northern Thailand, with a focus on behavioral and socioenvironmental determinants.

## Methods

### Study design and setting

This study employed a community-based, cross-sectional design to assess nutritional status using anthropometric indicators of growth and associated factors among ethnic minority preschool children. Data were collected between March and August 2023 in childcare centers (CCCs) located in Thoet Thai Subdistrict, Mae Fah Luang District, Chiang Rai Province, northern Thailand.

The study was conducted in Thoet Thai Subdistrict, Chiang Rai Province, a region inhabited by diverse ethnic minority groups, commonly referred to as hill tribes, who speak distinct languages and maintain unique cultural practices. Most communities reside in remote, agriculturally based areas with limited access to healthcare services and generally lower socioeconomic status than the ethnic Thai majority [21]. CCCs were selected as data collection sites because they serve as key community facilities that provide early education and care before children enter formal schooling. All CCCs operate under the supervision of the Thoet Thai Subdistrict Administrative Organization to ensure consistent childcare standards. CCCs in Thoet Thai Subdistrict were selected through a combination of convenience sampling and purposive outreach, considering ethnic diversity, geographic variation, accessibility, and each center’s willingness to participate. A total of 12 CCCs in the study area were contacted, of which 9 agreed to participate.

### Study participants

Children aged 2–5 years who were enrolled in the selected CCCs were eligible for participation if their parents or primary caregivers provided written informed consent. Children with conditions that could potentially affect the accuracy of anthropometric or dietary assessments—such as severe physical disabilities (e.g., cerebral palsy or congenital limb deformities), chronic illnesses affecting growth, or conditions influencing dietary intake—were excluded. Parents or primary caregivers provided written informed consent and actively participated in the study by completing the questionnaires and supporting all study procedures.

### Sample size and selection

Sample size calculation was performed to estimate an infinite population proportion, with nutritional status assessed using anthropometric indicators of growth as the primary outcome. The sample size was determined to achieve a statistical power of 80% and a significance level of 0.05. The required sample size was calculated using the standard formula for estimating a population proportion: n = [(Z_1−α_)^2^ ×p×(1 − p)​] / d^2^, where n represents the required sample size; Z is the Z-value corresponding to the desired confidence level (1.96 for 95% confidence); p is the estimated prevalence of stunting among children under five years of age in northern Thailand (0.154) [24]; and d is the margin of error (0.05). Based on this calculation, the minimum required sample size was 200 participants. To account for an anticipated 20% nonresponse or dropout rate, the target sample size was increased to 240 participants. A complete list of all eligible children was obtained from each childcare center, and participants were selected using a simple random sampling method through a lottery process to ensure that each child had an equal chance of selection. In this study, all eligible children and their caregivers agreed to participate, resulting in a final sample size of 260 participants, which exceeded the target sample size.

### Development of a questionnaire

A structured questionnaire was developed to collect information on factors associated with the nutritional status of hill tribe children. Questionnaire items were developed based on findings from previous studies and relevant literature to ensure that the content was appropriate and comprehensive for assessing these factors [20, 21, 25, 26]. The questionnaire was designed to assess caregiver- and family-related factors, categorized as socioenvironmental determinants, as well as dietary practices, categorized as behavioral determinants. The initial draft was reviewed by three experts in public health, pediatric nutrition, and community health research to evaluate content validity. The item-objective congruence (IOC) method was used to assess the relevance and appropriateness of each item, with acceptable item scores set at 0.5 or higher. Items scoring below 0.5 were revised or removed in accordance with expert recommendations. The final questionnaire achieved an overall IOC score of 0.83, indicating a high level of content validity. Following this revision process, a pilot study was conducted with 30 participants who shared characteristics similar to those of the target population. Based on feedback from the pilot study, the questionnaire was further refined to enhance its completeness and accuracy before implementation in the main study.

Dietary practices are defined as behaviors related to food consumption, including the frequency of food intake, types of foods consumed, eating habits, food choices, and other nutritional behaviors of preschool children [27]. The Food Frequency Questionnaire (FFQ) was selected as the most appropriate tool for this study, as it enables a systematic assessment of habitual dietary patterns and consumption frequency. The questionnaire was culturally adapted and validated prior to field data collection to ensure contextual relevance and measurement reliability. The FFQ used in this study was developed based on the Thai food-based dietary guidelines for preschool children [28] and commonly consumed foods among preschool-aged children in northern Thailand, as reported in the Database of Food Consumption of Thai People [29]. Food items were grouped according to nutritional characteristics and study objectives, with consumption frequency levels defined in accordance with previous research on preschool dietary behaviors [30–32]. The FFQ was pilot tested with 30 parents or primary caregivers in neighboring communities to refine food items and ensure consistency with local dietary practices prior to implementation in the main study. The final version of the FFQ comprised 33 food items, categorized into six groups: grains and starches, vegetables, fruits, meats and fish, milk and dairy products, and snacks and beverages.

Finally, the questionnaire was structured into four main sections to capture determinants of children’s nutritional status, including caregiver- and family-related factors (socioenvironmental determinants) and dietary practice factors (behavioral determinants). In Section I, child characteristics, including age, sex, ethnicity, and religion, were assessed. Section II collected information on family and household characteristics, such as parents’ marital status, occupation, and education, as well as details regarding other household members and overall household income. Section III focused on caregiving-related factors, encompassing the characteristics of the primary food preparer, caregiver nutritional knowledge, and meal preparation practices. The scoring system for caregiving components included two domains: (1) frequency of food preparation, categorized as low (0–8 points), moderate (9–18 points), and high (19–24 points); and (2) food preparation practices, classified as low (0–20 points), moderate (21–40 points), and high (41–60 points). Section IV addressed the dietary behaviors of preschool children, including the frequency and types of foods consumed, categorized into four levels: never, 1–2 times per week, 3–4 times per week, and daily. Food behavior scores were further assessed by food groups, such as carbohydrate intake, categorized into low (0–7 points), moderate (8–14 points), and high (15–21 points), and vegetable consumption, categorized into low (0–4 points), moderate (5–8 points), and high (9–12 points).

### Classification of the double burden of malnutrition in children

This study examined the double burden of malnutrition, which refers to the coexistence of undernutrition (stunting or wasting) and overnutrition (overweight) within the same population. The nutritional status of the children was assessed using the World Health Organization (WHO) Child Growth Standards to ensure international comparability and consistency with previous studies on malnutrition among preschool-aged children in Thailand. Children’s weight and height were measured in accordance with standard WHO protocols [2]. Body weight was measured using a digital weighing scale (HD-661 digital scale, TANITA), and height was measured using a portable stadiometer (Seca 217 portable stadiometer, SECA). All measurement instruments were calculated according to the manufacturers’ instructions prior to and throughout the data collection period. All anthropometric measurements were taken twice by trained personnel using calibrated equipment. If the two measurements differed by more than 0.1 kg for weight or 0.5 cm for height, a third measurement was obtained. The two closest values were averaged and used for analysis to ensure data accuracy and reliability. Nutritional status was subsequently determined on the basis of sex-specific Z-scores according to the WHO Child Growth Standards [33], with Z-scores calculated using WHO Anthro software version 3.2.2 [34]. Stunting was defined as a height-for-age Z-score (HAZ) below − 2 standard deviations (SDs), wasting as a weight-for-height Z-score (WHZ) below − 2 SDs, and overweight/obesity as a WHZ above + 2 SDs.

### Data collection

Trained research assistants and colleagues, who had received prior instruction on the study objectives, procedures, and data collection tools, conducted structured interviews with parents or primary caregivers at each childcare center. Practical training under expert supervision ensured their proficiency and accuracy in data collection. In most study areas, participants were able to communicate in standard Thai. However, in areas where local dialects or indigenous languages predominated, trained translators were employed. These translators were native speakers of the local languages and fluent in Thai, and they facilitated accurate communication of the interview questions to ensure full comprehension by participants. Parents or primary caregivers provided information on sociodemographic characteristics and their child’s frequency of consumption of each food item during the preceding month. Data were collected using paper-based questionnaires, and daily supervision by the research team was conducted to ensure data completeness and quality.

### Statistical analysis

The data were analyzed using IBM SPSS Statistics for Windows, Version 23.0 (IBM Corp., Armonk, NY, USA). Descriptive statistics were used to summarize participant characteristics. Frequencies and proportions were reported for categorical variables, whereas means and standard deviations were reported for continuous variables. All variables were initially examined using univariate logistic regression. Variables with a p-value < 0.20 in the univariate analysis, or those identified as relevant based on previous literature, were included in the multivariate binary logistic regression models. The binary outcomes included stunting, wasting, and overweight/obesity. Potential confounders considered in the analyses included child characteristics (age, sex, ethnicity, and religion), household characteristics (household members, income provider, number of siblings, and household income), and caregiver characteristics (occupation, marital status, education level, food preparation practices, and parental nutrition knowledge). Adjusted odds ratios (aORs) with 95% confidence intervals (CIs) were reported for each outcome. All statistical tests were two-tailed, and a p-value of less than 0.05 was considered statistically significant.

### Ethical considerations

The study protocol was reviewed and approved by the Human Research Ethics Committee of the Chiang Rai Provincial Public Health Office (CRPPHO No. 14/2023). Written informed consent was obtained from parents or primary caregivers prior to participation. To ensure voluntary participation and full comprehension, caregivers were provided with detailed information regarding the study objectives, data collection procedures, and the rights of both children and parents or primary caregivers to accept or decline participation without any impact on services provided by CCCs. In addition, verbal assent was obtained from the children prior to any measurements or data collection to confirm their willingness to participate. Parents or primary caregivers were required to be present and actively involved throughout all study procedures.

## Results

### Sociodemographic characteristics of preschool-aged minority children

A total of 260 children aged 2–5 years and their parents were included in the analysis. Of these, 145 (55.8%) were boys and 115 (44.2%) were girls, with a mean age of 3.5 ± 0.7 years. The prevalence of stunting, wasting, and overweight/obesity among the children was 22.3%, 20.8%, and 11.9%, respectively, indicating the coexistence of undernutrition and overnutrition within the same population. In terms of ethnicity, more than half of the children were Lahu (52.1%), followed by Tai Yai (18.8%), Akha (18.5%), and Yunnan Chinese (10.6%), reflecting the ethnic diversity of the study area. The majority of the children identified as Buddhist (56.2%), whereas 41.9% identified as Christian. Most parents were engaged in agriculture, trade, or daily wage labor (96.5%), and the majority were married (81.5%) and lived in extended households with more than five members (67.3%). Nearly half of the households (48.0%) reported a monthly income of less than 9,000 baht, and 48.5% of parents had no formal education, suggesting that socioeconomic vulnerability may influence child nutrition. Furthermore, parents were primarily responsible for food preparation (72.3%), and nearly half (47.7%) relied on online media as their main source of nutritional information (Table 1).

**Table 1:** Demographic and household characteristics of children and their families by malnutrition status (*n* = 260)

Variables	Category	TotalNumber (%)	Frequency (Percentage)					
			Stunting		Wasting		Overweight/obesity	
			Yes 58(22.3)	No202(77.7)	Yes54(20.8)	No206(79.2)	Yes31(11.9)	No229(88.1)
Child’s demographic characteristics								
Age Mean±S.D; 3.5 ± 0.7	2 years old	49(18.8)	19(38.8)	30(61.2)	9(18.4)	40(81.6)	5(10.2)	44(89.8)
	3 years old	137(52.7)	33(24.1)	104(75.9)	33(24.1)	104(75.9)	16(11.7)	121(88.3)
	4 years old	66(25.4)	5(7.6)	61(91.9)	10(15.1)	60(84.9)	8(12.1)	58(87.9)
	5 years old	8(3.1)	1(12.5)	7(87.5)	2(25.0)	6(75.0)	2(25.0)	6(75.0)
Sex	Boys	145(55.8)	34(23.4)	111(76.6)	26(17.9)	119(82.1)	10(8.7)	105(91.3)
	Girls	115(44.2)	24(20.9)	91(79.1)	28(24.3)	87(75.7)	21(14.5)	124(85.5)
Ethnicity	Akha	48(18.5)	10(20.8)	38(79.2)	12(25.0)	36(75.0)	6(12.5)	42(87.5)
	Lahu	137(52.1)	31(22.6)	106(77.4)	30(21.9)	107(78.1)	12(8.8)	125(91.2)
	Tai Yai	49(18.8)	10(20.4)	39(79.6)	9(18.4)	40(81.6)	7(14.3)	42(85.7)
	Yunnan Chinese	26(10.6)	7(26.9)	19(73.1)	3(11.5)	23(88.5)	6(23.1)	20(76.9)
Religion	Buddhism	146(56.2)	38(26.0)	108(74.0)	28(19.2)	118(80.8)	19(13.0)	127(87.0)
	Christianity	109(41.9)	17(15.6)	92(84.4)	22(20.2)	87(79.8)	10(9.2)	99(90.8)
	Ancient/others	5(1.9)	3(60.0)	2(40.0)	4(80.0)	1(20.0)	2(40.0)	3(60.0)
Family demographic characteristics								
Marital status of parents	Married	212(81.5)	48(22.6)	164(77.4)	45(21.2)	167(78.8)	25(11.8)	187(88.2)
	Divorce/ widow	48(18.5)	10(20.8)	38(79.2)	9(18.8)	39(81.2)	6(12.5)	42(87.5)
Occupation of parents	Unemployed	5(1.9)	18(24.0)	57(76.0)	0(0.0)	5(100.0)	1(20.4)	4(80.0)
	Agriculture/mer- chant/daily hire	251(96.5)	39(21.4)	143(78.6)	54(21.5)	197(78.5)	29(11.6)	222(88.4)
	Government officer	4(1.5)	1(33.3)	2(66.7)	0(0.0)	4(100.0)	1(25.0)	3(75.0)
Purchased ready-to-eat food	Yes	162(62.3)	38(23.5)	124(76.5)	18(18.4)	80(81.6)	22(13.6)	140(86.4)
	No	98(37.7)	20(20.4)	78(79.6)	36(22.2)	126(77.8)	9(9.2)	89(90.8)
Education level of parents*	Illiterate	126(48.5)	29(23.0)	97(77.0)	24(19.0)	102(81.0)	13(10.3)	113(89.7)
	Primary school	49(18.9)	11(22.4)	38(77.6)	12(24.5)	37(75.5)	7(14.3)	42(85.7)
	Secondary school	69(26.5)	15(21.7)	54(78.3)	14(20.3)	55(79.7)	9(13.0)	60(87.0)
	Vocational certificate	6(2.3)	2(33.3)	4(66.7)	3(50.0)	3(50.0)	1(16.7)	5(83.3)
	High vocational certificate (Diploma)	4(1.5)	0	4(100.0)	1(25.0)	3(75.0)	0	4(100.0)
	Bachelor’s degree	6(2.3)	1(16.7)	5(83.3)	0	1(16.7)	1(16.7)	5(83.3)
Householdsmembers	2-4 persons	98(37.7)	17(17.4)	81(82.6)	18(18.4)	80(81.6)	17(17.3)	81(82.7)
	5 persons	56(21.5)	15(26.8)	41(73.2)	15(26.8)	41(73.2)	7(12.5)	49(87.5)
	>6 persons	106(40.8)	26(24.5)	80(75.5)	21(19.8)	85(80.2)	7(6.6)	99(93.4)
Number of siblings	None	24(9.2)	6(25.0)	18(75.0)	7(29.2)	17(70.8)	4(16.7)	20(83.3)
	1-2 persons	152(58.5)	35(23.0)	117(77.0)	25(16.5)	127(83.6)	21(13.8)	131(86.2)
	>3 person	84(32.4)	18.0(21.4)	66(78.6)	21(25.0)	63(75.0)	7(8.3)	77(91.7)
Household income	<9,000 baht	125(48.0)	27(21.6)	98(78.4)	29(23.2)	96(76.8)	16(12.8)	109(87.2)
	>9,000 baht	135(52.0)	31(23.0)	104(77.0)	25(18.5)	110(81.5)	15(11.1)	120(88.9)
Characteristics and nutrition knowledge of the food preparation								
Food preparer	Mother/Father	188(72.3)	44(23.4)	144(76.6)	46(24.5)	142(75.5)	21(11.2)	167(88.8)
	Others	72(27.7)	14(19.4)	58(80.6)	8(11.1)	64(88.9)	10(13.9)	62(86.1)
Home-pre pared meals	Yes	256(98.5)	57(22.3)	199(77.7)	53(20.7)	203(79.3)	30(11.7)	226(88.3)
	No	4(1.5)	1(25.0)	3(75.0)	1(25.0)	3(75.0)	1(25.0)	3(75.0)
Purchased ready-to-eat food	Yes	162(62.3)	38(23.5)	124(76.5)	18(18.4)	80(81.6)	22(13.6)	140(86.4)
	No	98(37.7)	20(20.4)	78(79.6)	36(22.2)	126(77.8)	9(9.2)	89(90.8)
Receiving nutrition knowledge of parents	Yes	166(63.8)	41(24.7)	125(75.3)	34(20.5)	132(79.5)	20(12.1)	146(87.9)
	No	94(36.2)	17(18.1)	77(81.9)	20(21.3)	74(78.7)	11(11.7)	83(88.3)
Sources of nutrition knowledge	Online media	124(47.7)	26(21.1)	98(78.9)	21(16.9)	103(83.1)	15(12.1)	109(87.9)
	Television	34(13.1)	12(35.3)	22(64.7)	9(26.5)	25(73.5)	7(20.6)	27(79.4)
	Healthcare personnel	18(6.9)	4(22.2)	14(77.8)	5(27.8)	13(72.2)	2(11.1)	16(88.9)
	Nutritionists	7(2.7)	0	7(100.0)	1(14.3)	6(85.7)	0	7(100.0)
	Village health volunteers	26(10.0)	8(30.8)	18(69.7)	8(30.8)	18(69.7)	2(7.7)	24(92.3)
	Teachers	45(17.3)	8(17.7)	37(82.3)	8(17.8)	37(82.3)	4(8.9)	41(91.1)
	Printed materials	6(2.3)	0	6(100.0)	2(33.3)	4(66.7)	1(16.7)	5(83.3)

### Dietary practices of preschool-aged ethnic minority children

**Fig. 1:**
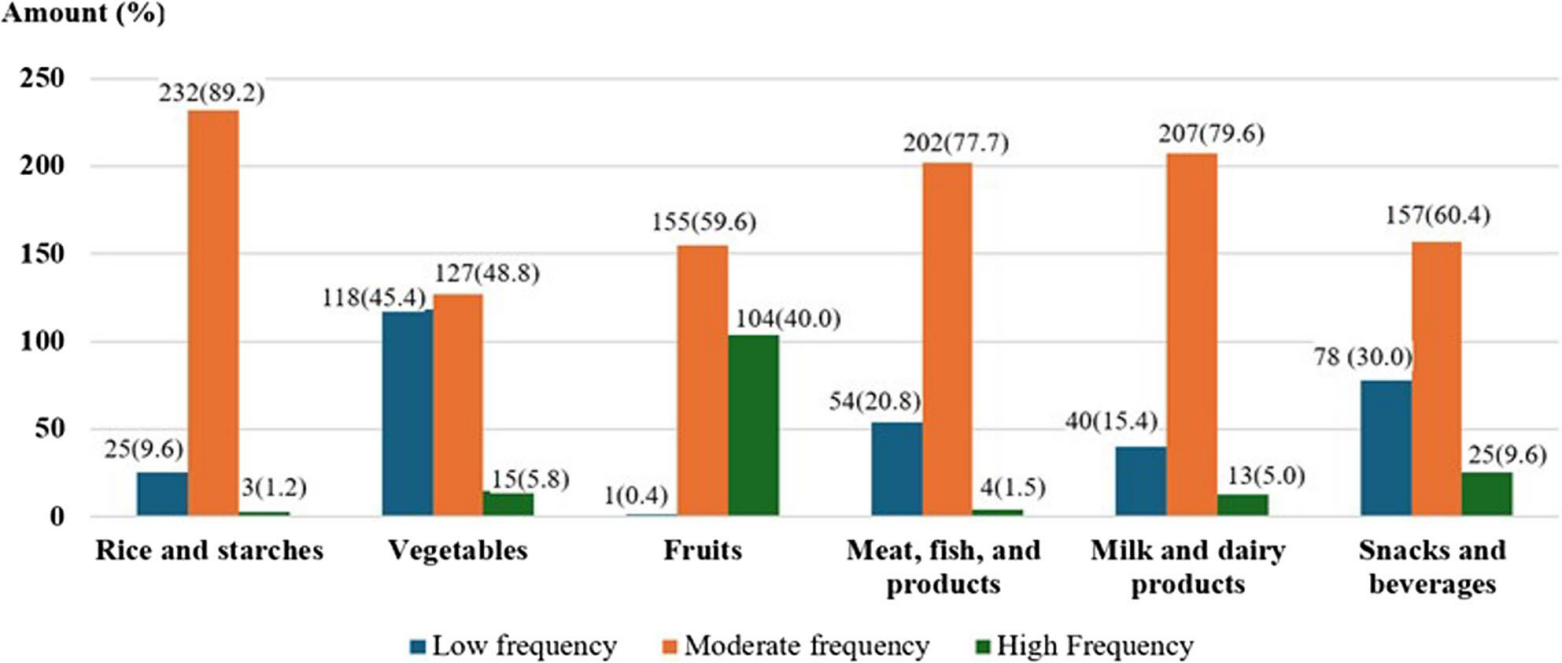
Food consumption frequency categorized by food group. Note: Low frequency = less than once per week; Moderate = 1–2 times per week; High = more than twice per week. * The “milk” category includes all types of milk, covering both cow’s milk and formula

Figure 1 illustrates the frequency of food consumption by food group. Food items were categorized according to consumption frequency as low (< once per week), moderate (1–2 times per week), and high (> twice per week). The analysis indicated that preschool children most frequently consumed foods from the grains and starches group, followed by the milk and dairy products, meat and fish, and snacks and beverages groups. In contrast, the consumption of vegetables and fruits remained relatively low. This pattern suggests a dietary imbalance, with a potential risk of inadequate micronutrient intake despite sufficient energy consumption from staple and protein-rich foods. Detailed information on the frequency of food consumption by food group is provided in the Additional files.

### Factors associated with the double burden of malnutrition

Tables 2 and 3 present the results of the univariate and multivariate logistic regression analyses examining the associations between the double burden of malnutrition and potential contributing factors. The outcome variables included stunting, wasting, and overweight/obesity, while the independent variables encompassed socioenvironmental factors and dietary behavior factors. The findings indicated that stunting was significantly associated with the frequency of rice and starch consumption. Children who consumed these foods at moderate to high frequencies had markedly higher odds of being stunted compared with those who consumed them infrequently (cOR = 7.69; 95% CI: 1.02–58.08, *p* = 0.048; aOR = 9.03; 95% CI: 1.18–69.05, *p* = 0.034), suggesting that an overreliance on starchy foods may contribute to growth faltering due to limited dietary diversity. Wasting was significantly associated with the identity of the meal preparer. Children whose meals were prepared by nonparental caregivers had more than twice the odds of wasting compared with those whose meals were prepared by parents (cOR = 2.59; 95% CI: 1.16–5.81, *p* = 0.021; aOR = 2.51; 95% CI: 1.12–5.64, *p* = 0.026), indicating the influence of caregiving practices and feeding responsibility on acute undernutrition. Overweight/obesity was significantly associated with low vegetable consumption and maternal income contribution. Children who consumed vegetables infrequently had more than twice the odds of being overweight or obese (cOR = 2.43; 95% CI: 1.11–5.31, *p* = 0.026; aOR = 2.35; 95% CI: 1.07–5.20, *p* = 0.034). Similarly, children from households in which mothers were the primary income providers had more than threefold higher odds of overweight/obesity (cOR = 3.22; 95% CI: 1.28–8.13, *p* = 0.013; aOR = 3.14; 95% CI: 1.23–8.04, *p* = 0.017), suggesting that limited vegetable intake and maternal employment-related factors may contribute to excessive energy intake and an increased risk of obesity among preschool children. No other variables showed statistically significant associations with any component of the double burden of malnutrition.

**Table 2:** Demographic and socioenvironmental characteristics associated with a double burden of malnutrition (univariate/multivariate logistic regression)

Variables	Category	Stunting				Wasting				Overweight/Obesity			
		Univariatelogistic regression		Multivariatelogistic regression		Univariatelogistic regression		Multivariatelogistic regression		Univariatelogistic regression		Multivariatelogistic regression	
		cOR (95% CI)	p-value	aOR (95% CI)	p value	cOR (95% CI)	p value	aOR(95% CI)	p value	cOR(95% CI)	p value	aOR(95% CI)	p value
Age:	³ 4 years old	1.00		1.00		1.00				1.00			
	3 years old	3.60(1.43-9.04)	0.007*	3.96(1.6–10.0)	<0.001*	1.64(0.79-3.41)	0.185	-	-	0.85(0.36-1.97)	0.699	-	-
	2 years old	7.18(2.6-19.77)	<0.001*	7.65(2.8–21.3)	0.004*	1.16(0.45-3.01)	0.756	-	-	0.73(0.23-2.74)	0.584	-	-
Sex	Girls	1.00				1.00				1.00			
	Boys	1.16(0.64-2.10)	0.620	-	-	1.47(0.81-.69)	0.207	-	-	1.78(0.80-3.94)	0.157		
Ethnicity	Akha	1.00				1.00				1.00			
	Lahu	1.11(0.50-2.48)	0.797	-	-	0.84(0.39-1.81)	0.659	-	-	0.67(0.24-1.90)	0.454	-	-
	Tai Yai	0.97(0.36-2.61)	0.959	-	-	0.68(0.26-1.79)	0.429	-	-	1.17(0.36-3.76)	0.796	-	-
	Yunnan Chinese	1.40(0.46-4.26)	0.553	-	-	0.39(0.10-1.54)	0.179	-	-	2.10(0.60-7.33)	0.245	-	-
Religion	Buddhism	1.00				1.00				1.00			
	Christianity/Ancient/others	0.61(0.33-1.11)	0.105	-	-	1.25(0.68-2.27)	0.475	-	-	0.79(0.37-1.70)	0.540	-	-
Occupation of parents	Government officer	1.00				1.00				1.00			
	Agriculture/merchant /daily hire	0.55(0.04-6.17)	0.624	-	-	0.48(0.04-5.40)	0.549	-	-	0.39(0.04-3.89)	0.424	-	-
	Unemployed	0.63(0.05-7.38)	0.714	-	-	0.63(0.05-7.38)	0.714	-	-	0.75(0.03-17.51)	0.858	-	-
Marital status of parents	Married	1.00				1.00				1.00			
	Divorce/ widow	0.90(0.42-1.94)	0.786	-	-	0.57(0.27-1.20)	0.137	-	-	1.07(0.41-2.76)	0.891	-	-
Householdsmembers	2-4 persons	1.00				1.00				1.00			
	5 persons	1.69(0.72-3.92)	0.226	-	-	1.69(0.72-3.92)	0.226	-	-	0.58(0.19-1.71)	0.321	-	-
	>6 persons	1.46(0.73-2.91)	0.285	-	-	1.21(0.60-2.46)	0.591	-	-	0.53(0.23-1.22)	0.136	-	-
Income provider	Father	1.00				1.00				1.00		1.00	
	Mother	1.09(0.44-5.07)	0.853	-	-	0.25(0.06-1.11)	0.068	-	-	3.22(1.28-8.13)	0.013*	3.14(1.23-8.04)	0.017*
	Other	0.72(0.39-1.56)	0.569	-	-	0.95(0.34-2.70)	0.926	-	-	0.38(0.05-2.99)	0.384	0.40(0.05-3.15	0.384
Number of siblings	None	1.00				1.00				1.00			
	1-2 persons	1.01(0.35-2.97)	0.981	-	-	0.52(0.18-1.47)	0.218	-	-	0.38(0.10-1.39)	0.144	-	-
	>3 person	0.92(0.31-2.78)	0.887	-	-	0.90(0.32-2.54)	0.843	-	-	0.74(0.23-2.41)	0.614	-	-
Household income	>9,000 baht	1.00				1.00				1.00			
	<9,000 baht	1.06(0.60-1.95)	0.844	-	-	1.06(0.60-1.95)	0.844	-	-	1.07(0.49-2.35)	0.860	-	-
Education level of parents	Secondary school or higher	1.00				1.00				1.00			
	Primary school	1.05(0.44-2.50)	0.908	-	-	1.19(0.51-2.78)	0.685	-	-	1.31(0.47-3.64)	0.609	-	-
	No formal education/ Illiterate	1.10(0.57-2.12)	0.772	-	-	0.87(0.44-1.70)	0.678	-	-	0.82(0.35-1.92)	0.640	-	-
Food preparer	Mother/Father	1.00				1.00		1.00		1.00			
	Others	1.27(0.65-2.48)	0.493	-	-	2.59(1.16-5.81)	0.021*	2.51(1.12-5.64)	0.026*	0.78(0.35-1.75)	0.546	-	-
Home-pre pared meals	Yes	1.00				1.00				1.00			
	No	1.16(0.12-11.4)	0.896	-	-	1.28(0.13-12.52)	0.834	-	-	2.51(0.25-24.92)	0.432	-	-
Purchased ready-to-eat food	No	1.00				1.00				1.00			
	Yes	1.20(0.65-2.20)	0.567	-	-	1.27(0.68-2.39)	0.458	-	-	1.55(0.69-3.53)	0.292	-	-
Receiving Nutrition knowledge of parents	Yes	1.00				1.00				1.00			
	No	0.67(0.36-1.26)	0.220	-	-	1.05(0.56-1.95)	0.879	-	-	0.97(0.44-2.12)	0.934	-	-

* Statistically significant at p < 0.05

*cOR*  crude odds ratio, *aOR*  adjusted odds ratio, *CI*  confidence interval

**Adjusted for the variables listed for each outcome in the multivariate logistic regression models

Stunting: Adjusted for child sex, ethnicity, religion, occupation of parents, marital status of parents, household members, income provider, number of siblings, household income, education level of parents, food preparer, receiving nutrition knowledge of parents

Wasting: Adjusted for child age, sex, ethnicity, religion, occupation of parents, marital status of parents, household members, income provider, number of siblings, household income, education level of parents, and nutritional knowledge of parents

Overweight/Obesity: Adjusted for child sex, ethnicity, religion, occupation of parents, marital status of parents, household members, number of siblings, household income, education level of parents, food preparation, and receiving nutrition knowledge from parents

***Variables included in the adjusted models were selected on the basis of univariate analysis (p < 0.20) and literature review to ensure relevance and minimize bias

**Table 3:** Dietary practices associated with the double burden of malnutrition among preschool-aged ethnic minority children (univariate and multivariate logistic regression analysis)

Variables	Category	Stunting				Wasting				Overweight/Obesity			
		Univariatelogistic regression		Multivariatelogistic regression		Univariatelogistic regression		Multivariatelogistic regression		Univariatelogistic regression		Multivariatelogistic regression	
		cOR (95% CI)	P value	aOR (95% CI)	p value	cOR (95% CI)	p value	aOR (95% CI)	p value	cOR (95% CI)	p value	aOR (95% CI)	p value
Food consumption frequency in food groups													
Rice and starches group	Low	1.00				1.00				1.00			
	High/Moderate	7.69(1.02-58.08)	0.048*	9.03(1.18-69.05)	0.034*	1.05(0.38-2.95)	0.921	-	-	1.62(0.36-7.23)	0.528	-	-
Vegetable group	High/Moderate	1.00				1.00				1.00		1.00	
	Low	1.06(0.59-1.91)	0.840	-	-	0.95(0.52-1.74)	0.876	-	-	2.43(1.11-5.31)	0.026*	2.35(1.07-5.20)	0.034*
Fruit group	High/Moderate	1.00				1.00				1.00			
	Low	N/A	N/A	-	-	N/A	N/A	-	-	N/A	N/A	-	-
Meat, fish, and products group	High/Moderate	1.00				1.00				1.00			
	Low	1.89(0.64-2.57)	0.474	-	-	1.45(0.72-2.92)	0.296	-	-	1.13(0.46-2.78)	0.791	-	-
Milk and dairy products group	High/Moderate	1.00				1.00				1.00			
	Low	0.70(0.29-1.68)	0.429	-	-	1.13(0.50-2.54)	0.769	-	-	1.73(0.69-4.34)	0.241	-	-
Snacks and beverages group	Low	1.00				1.00				1.00			
	High/Moderate	1.30(0.67-2.51)	0.436	-	-	1.45(0.73-2.89)	0.287	-	-	1.27(0.54-2.97)	0.588	-	-

* Statistically significant at p < 0.05

*cOR*  crude odds ratio, *aOR*  adjusted odds ratio, *CI*  confidence interval

**Adjusted for the variables listed for each outcome in the multivariate logistic regression models

Stunting: Adjusted for vegetable group, fruit group, meat group, milk and dairy products group, snacks and beverages group

Overweight/Obesity: Adjusted for the rice and starch group, fruit group, meat group, milk and dairy product group, and snacks and beverages group

Variables included in the adjusted models were selected on the basis of univariate analysis (p < 0.20) and literature review to ensure relevance and minimize bias

## Discussion

This community-based cross-sectional study examined the associations between the double burden of malnutrition and its behavioral and socioenvironmental determinants among preschool-aged ethnic minority children (2–5 years) residing in Thoet Thai Subdistrict, Mae Fah Luang District, Chiang Rai Province, northern Thailand. In this study population, the double burden of malnutrition was defined as the coexistence of undernutrition (stunting and wasting) and overnutrition (overweight and obesity). Globally, approximately 22.0% of children under five years of age are stunted and 6.7% are wasted [6], while national data from Thailand in 2020 reported lower prevalence rates of stunting (12.3%), wasting (7.7%), and overweight/obesity (9.2%) [35]. In contrast, the prevalence observed in the present study was substantially higher, with 22.3% of children being stunted, 20.8% wasted, and 11.9% overweight/obese. These findings indicate a disproportionate nutritional burden among ethnic minority preschool children in this rural setting, with the prevalence of stunting nearly twice the national average. Comparable patterns have been reported in other ethnic minority populations in northern Thailand. For example, a study conducted among ethnic preschool children in Chiang Mai Province documented prevalence rates of 16.3% for stunting, 10.9% for wasting, and 19.7% for overweight/obesity [17], suggesting that ethnic minority children in mountainous and remote areas experience persistent nutritional vulnerabilities. Age-specific analyses in present study further revealed distinct nutritional patterns across early childhood. Children aged two years reported the highest prevalence of stunting (38.8%) and wasting (18.4%), whereas three-year-old children showed continued high levels of stunting (24.1%) alongside an emerging prevalence of overweight (11.7%). In contrast, children aged four years and older demonstrated lower rates of stunting (8.1%) and wasting (16.2%) but a higher prevalence of overweight and obesity (13.5%). The relatively higher prevalence of stunting observed among younger children may be attributed to suboptimal complementary feeding practices and adverse early-life nutritional exposures during the first two years of life [36, 37]. Inadequate dietary diversity delayed or inappropriate complementary feeding, and increased susceptibility to infections and environmental stressors during this critical growth period may collectively impair linear growth and increase the risk of early childhood stunting. As children transition into later preschool years, a gradual shift from undernutrition toward overweight and obesity becomes apparent. This pattern is consistent with the nutrition transition described in low- and middle-income countries, where rapid changes in dietary behaviors and food environments coexist with persistent early-life undernutrition [38–40], reflecting the coexistence of undernutrition and overnutrition within the same population and illustrating the double burden of malnutrition.

Stunting remains a major public health concern in low- and middle-income countries because of its multifactorial etiology and long-term consequences for physical growth, cognitive development, and health [41, 42]. Adequate complementary feeding alongside breastfeeding is critical from six months of age to meet increasing nutrient requirements [43]. In the present study, stunting among children aged 2–3 years was strongly associated with frequent consumption of staple starchy foods, whereas no associations were observed with other food groups. Children with moderate to high intake of carbohydrate-rich staples were approximately nine times more likely to be stunted than those with low intake (OR = 9.03, 95% CI: 1.18–69.05). This finding may indicate limited dietary diversity among children who rely heavily on carbohydrate-based foods, for example sticky rice, instant noodles, and rice porridge. In the study area, sticky rice is the primary carbohydrate provided by caregivers and is often consumed in large quantities with relatively small portions of side dishes (i.e., consuming more rice but less accompaniment), which may consequently result in an inadequate intake of other essential nutrients. Excessive intake of starchy staples without sufficient protein and micronutrient sources may lead to inadequate nutrient intake, which is required for optimal linear growth [44, 45]. Findings from other populations have been mixed. A study among Iranian schoolgirls aged 6–12 years reported a positive association between carbohydrate intake and height-for-age Z scores (HAZ) [46]. Differences from the present study may reflect contextual factors, including participant age, sex, and the categorization of carbohydrate containing foods. The Iranian study classified carbohydrates by physical form (liquid vs. solid), whereas the current study categorized intake into staple starchy foods, snacks, and beverages, which may explain the inconsistencies in the findings. Other studies have reported no significant association between carbohydrate intake and stunting in preschool children [42]. Moreover, several studies have reported that stunted children consistently consume less energy, protein, fat, iron, and vitamin A than nonstunted children [42, 47–49]. These findings underscore that imbalances in macronutrient and micronutrient intake may increase the risk of stunting even when total energy intake is sufficient [50]. Overall, the results highlight that although carbohydrate-rich foods are important sources of energy, excessive dependence on staple starches in the absence of adequate dietary diversity may compromise nutritional quality and hinder optimal growth among preschool-aged children.

In the present study, wasting was more prevalent among children whose meals were prepared by nonparental caregivers, underscoring the importance of socioenvironmental factors in child nutrition. Parents and primary caregivers play a crucial role in determining children’s nutritional status, as their nutrition-related knowledge, attitudes, and feeding practices directly influence both the quality and quantity of foods provided, thereby affecting growth and malnutrition risk [51]. Consistent with this evidence, a study from Canada reported that active parental involvement in meal preparation is associated with lower child nutrition risk scores, highlighting the protective role of appropriate caregiver feeding practices [52]. In the present study setting, nonparental caregivers were predominantly grandmothers who were older adults and often had limited formal education. In rural ethnic minority communities, intergenerational caregiving is common, with grandparents frequently assuming primary responsibility for child-rearing and meal preparation [53–55]. While such arrangements can provide emotional support and household stability, they may also pose nutritional challenges. Several studies suggest that grandparents may rely on snacks or sweetened beverages rather than nutritionally balanced meals, partly due to limited nutrition knowledge and insufficient awareness of age-appropriate dietary requirements [56–58]. Moreover, child-directed feeding practices, which prioritize children’s immediate preferences over nutritional adequacy, are frequently observed in intergenerational caregiving contexts [58–60]. Additional contextual factors may further contribute to the observed association. As this cross-sectional study was conducted during a relatively short period that coincided with farming seasons, some grandparents may have had limited time to prepare regular or balanced meals due to agricultural workloads [61, 62]. Taken together, these findings suggest that nonparental caregiving, particularly by elderly grandparents with constrained resources and nutrition knowledge, may increase the risk of wasting among children in this population. Strengthening nutrition education and support for intergenerational caregivers may therefore be essential for improving child nutritional outcomes in similar settings.

Overweight and obesity among preschool-aged children in this study were significantly associated with low fruit consumption and maternal identification as the primary household income earner. During the preschool years, children gradually gain autonomy over food choices and begin to establish dietary patterns that may persist into later life [63, 64]. This developmental period also coincides with adiposity rebound, a physiological phase characterized by increasing fat accumulation and rising body mass index (BMI), which has been linked to a heightened risk of later obesity and metabolic disorders [65–67]. In line with these developmental processes, previous studies have consistently reported that low fruit and vegetable consumption is associated with an increased risk of overweight and obesity among preschool-aged children [68–72], supporting the findings observed in the present study. The low intake of fruits and vegetables observed in this population reflects broader national dietary trends in Thailand, where consumption of these food groups has declined across all age groups, particularly among young children [73]. Preschool-aged children consume an average of approximately 150 g of fruits and vegetables per day, far below national dietary recommendations, with most children consuming less than 30% of the recommended intake [74, 75]. Beyond individual preferences, the food environment—including availability, affordability, and culturally embedded dietary practices—plays a critical role in shaping children’s fruit and vegetable consumption [76]. Maternal socioeconomic roles may further influence children’s dietary behaviors. Mothers, who are often the primary caregivers and meal preparers, play a central role in shaping children’s eating patterns. Evidence from Taiwan suggests that mothers generally demonstrate healthier feeding practices than fathers or grandparents, indicating both their importance and potential for targeted support [77]. However, when mothers are the primary income earners, competing work demands may limit time for meal planning and food preparation, increase reliance on convenience or energy-dense foods, and ultimately lower overall diet quality [78, 79]. Although maternal employment was not directly associated with wasting in this study, reduced parental involvement in daily feeding routines may still compromise dietary diversity and contribute to excessive energy intake among preschool children. Notably, despite their pivotal role, most parents in this study reported relying primarily on online media, such as social media platforms, websites, and online news, as their main sources of nutrition information rather than consulting qualified nutrition professionals. These sources are often unregulated and may disseminate inaccurate or misleading information, potentially reinforcing inappropriate feeding practices. Collectively, these findings highlight gaps in local policy implementation, limited access to reliable nutrition information, and persistent inequities in nutrition education services. Addressing these challenges requires strengthening community-based nutrition education programs, integrating qualified nutrition professionals into primary healthcare and early childhood settings, and implementing school- and community-based interventions that promote fruit and vegetable consumption using culturally appropriate and context-specific communication strategies.

A major strength of this study lies in its focus on preschool-aged ethnic minority children in northern Thailand, a hard-to-reach population that has been consistently underrepresented in previous research. Data collection across multiple childcare centers located in both lowland and remote hill areas enabled a comprehensive assessment of dietary behaviors and socioenvironmental factors across diverse community contexts. In addition, the use of WHO growth standard Z-scores provided a scientifically robust and internationally comparable assessment of children’s nutritional status across age and sex groups. Collectively, these strengths allow the present findings to contribute valuable international evidence on the complex interactions between socioenvironmental and behavioral determinants of malnutrition among ethnic minority preschool children. The sociocultural and geographic characteristics of these communities, such as cultural diversity and limited access to healthcare and nutrition services, are also shared by many marginalized and rural populations in other low- and middle-income countries, enhancing the broader relevance of the findings. Nevertheless, several limitations should be acknowledged. First, the cross-sectional design precludes causal inference between the identified factors and nutritional outcomes. Second, dietary intake was assessed using a caregiver-reported FFQ, which is subject to recall bias and does not provide precise quantitative estimates of actual food consumption. Third, the use of non-probability sampling methods, combining convenience and purposive approaches, may limit the generalizability of the findings beyond the study area due to potential selection bias. As such, the results should be interpreted with caution when extrapolated to other ethnic minority groups or populations with different sociodemographic and cultural contexts. Future research should employ longitudinal or intervention-based study designs to better establish causal relationships between dietary practices, caregiver-related factors, and child nutritional outcomes. Incorporating more objective dietary assessment methods, such as repeated 24-hour dietary recalls or direct observation, would improve the accuracy of dietary intake measurements. Additionally, distinguishing energy-dense, nutrient-poor foods as separate categories within dietary assessment tools may provide greater insight into their specific contributions to malnutrition. Expanding studies to include a wider range of ethnic groups and geographic regions using probability-based sampling across multiple provinces in Thailand would further enhance representativeness, strengthen external validity, and support a more comprehensive understanding of malnutrition among preschool-aged children.

## Conclusions

This study highlights the critical role of family practices and the caregiving environments in shaping the double burden of malnutrition among preschool-aged ethnic minority children. Effective interventions should focus on enhancing caregiver nutrition literacy, supporting maternal engagement in balanced caregiving alongside employment, integrating qualified nutrition professionals into local childcare systems, and ensuring equitable access to diverse and nutritious foods. Addressing these household and socioenvironmental determinants is essential to prevent both undernutrition and overnutrition and to promote healthy growth and development in this vulnerable population.

## Supplementary Information

Supplementary Material 1.

## Data Availability

All the data generated or analyzed during this study are included in this published article and its supplementary information files.

## References

[1] Pinto J, Da Z, Ximenes C, de Jesus A. The role of nutrition in children’s growth and development at early age: systematic review. Int J Res Sci Technol. 2023;14(1):1–10.

[2] World Health Organization. Training course on child growth assessment. https://www.who.int/publications/i/item/9789241595070 (2008). Accessed 20 Sep 2025.

[3] Jehn M, Brewis A. Paradoxical malnutrition in mother–child pairs: untangling the phenomenon of over- and undernutrition in underdeveloped economies. Econ Hum Biol. 2009;7(1):28–35. 19246260 10.1016/j.ehb.2009.01.007

[4] Doak CM, Adair LS, Bentley M, Monteiro C, Popkin BM. The dual burden household and the nutrition transition paradox. Int J Obes (Lond). 2005;29(1):129–36. 15505634 10.1038/sj.ijo.0802824

[5] Popkin BM, Corvalan C, Grummer-Strawn LM. Dynamics of the double burden of malnutrition and the changing nutrition reality. Lancet. 2020;395(10217):65–74. 31852602 10.1016/S0140-6736(19)32497-3 PMC7179702

[6] United Nations Children’s Fund (UNICEF), World Health Organization, International Bank for Reconstruction and Development/The World Bank. Levels and trends in child malnutrition: UNICEF/WHO/The World Bank Group joint child malnutrition estimates: key findings of the 2021 edition. 21st ed. https://www.who.int/publications/i/item/9789240025257 (2021). Accessed 20 Sep 2025.

[7] Azriani D, Masita, Qinthara NS, Yulita IN, Agustian D, Zuhairini Y, Dhamayanti M. Risk factors associated with stunting incidence in underfive children in Southeast Asia: a scoping review. J Health Popul Nutr. 2024;43(1):1–14. 39468694 10.1186/s41043-024-00656-7 PMC11520880

[8] National Statistical Office of Thailand, UNICEF. Thailand Multiple Indicator Cluster Survey 2022, Survey Findings Report. Bangkok: National Statistical Office of Thailand; 2023.

[9] Ministry of Public Health. Thailand. Nutritional status of children aged 3–5 years. 2023.

[10] Alem AZ, Yeshaw Y, Liyew AM, Tessema ZT, Worku MG, Tesema GA, Alamneh TS, Teshale AB, Chilot D, Ayalew HG. Double burden of malnutrition and its associated factors among women in low and middle income countries: findings from 52 nationally representative data. BMC Public Health. 2023;23:1. 37537530 10.1186/s12889-023-16045-4 PMC10398981

[11] Mekonnen S, Birhanu D, Menber Y, Gebreegziabher ZA, Belay MA. Double burden of malnutrition and associated factors among mother–child pairs at household level in Bahir Dar City, Northwest Ethiopia: community based cross-sectional study design. Front Nutr. 2024;11:2024. 10.3389/fnut.2024.1340382 PMC10912183 38445209

[12] Gebremichael B, Abera A, Biadgilign S, Baye K, Zhou SJ, Haile D. Double burden of malnutrition among under-five children in Eastern and Southern African countries. Sci Rep. 2025;15:1. 40169590 10.1038/s41598-025-87144-y PMC11961756

[13] Yigezu M, Oumer A, Damtew B, Birhanu D, Getaye Workie S, Hamza A, Atle A, Kebede N. The dual burden of malnutrition and its associated factors among mother-child pairs at the household level in Ethiopia: an urgent public health issue demanding sector-wide collaboration. PLoS ONE. 2024;19(11):e0307175. 39495734 10.1371/journal.pone.0307175 PMC11534222

[14] Talukder A, Kelly M, Gray D, Sarma H. Prevalence and trends of double burden of malnutrition at household-level in South and Southeast Asia. Discover Public Health. 2024;21:1.

[15] Kiosia A, Dagbasi A, Berkley JA, Wilding JPH, Prendergast AJ, Li JV, Swann J, Mathers JC, Kerac M, Morrison D, et al. The double burden of malnutrition in individuals: identifying key challenges and re-thinking research focus. Nutr Bull. 2024;49(2):132–45. 38576109 10.1111/nbu.12670

[16] Benedict L, Hong SA, Winichagoon P, Tejativaddhana P, Kasemsup V. Double burden of malnutrition and its association with infant and young child feeding practices among children under five in Thailand. Public Health Nutr. 2021;24(10):3058–65. 33054885 10.1017/S1368980020003304 PMC9884786

[17] Janpeang J, Suwannapoom C, Anukunwathaka N. Nutritional status and related factors among ethnic preschool children in Northern Thailand: a cross-sectional study. Child Health Nurs Res. 2022;28(3):176–86. 35953067 10.4094/chnr.2022.28.3.176 PMC9371800

[18] Ansuya, Nayak BS, Unnikrishnan B, George A, N SY, Mundkur SC, Guddattu V. Risk factors for malnutrition among preschool children in rural Karnataka: a case-control study. BMC Public Health. 2018;18:1. 10.1186/s12889-018-5124-3 PMC5828120 29482540

[19] Center for Maternal and Child Survival (CMS). Advancing health equity in rural, tribal, and geographically isolated communities. 2022. Available from: www.cms.gov/files/document/fy-2023-advancing-rural-health-508.pdf. Accessed 20 Sep 2025.

[20] Medicine Division; Board on Population Health and Public Health Practice; Committee on Community-Based Solutions to Promote Health Equity in the United States, Health M. In: Baciu A, Geller A, Weinstein JN, editors. Communities in action: pathways to health equity. Washington (DC): National Academies; 2017. 28418632

[21] Apidechkul T, Laingoen O, Suwannaporn S. Inequity in accessing health care service in Thailand in 2015: a case study of the hill tribe people in Mae Fah Luang District, Chiang Rai, Thailand. J Health Res. 2017;30(1):67–71.

[22] Apidechkul T, Siriyaporn Sittisarn T, Ruanjai. Health situation of Akha hill tribe in Chiang Rai Province, Thailand. J Public Health Dev. 2016;14(1):77–97.

[23] Perrone C, Kanthawang N, Cheah PY. A hill tribe community advisory board in Northern Thailand: lessons learned one year on. Int J Equity Health. 2024;23(1):1–10. 39558319 10.1186/s12939-024-02323-z PMC11574996

[24] Waleewong O, Yueayai K. Patterns of socioeconomic inequities in SDGs relating to children’s well-being in Thailand and policy implications. Int J Environ Res Public Health. 2022;19(20):13626. 36294206 10.3390/ijerph192013626 PMC9603103

[25] Sroywong D, Wungrath J, Thongprachum A. Factors associated with stunting among hill tribe preschool children in Thailand. J Med Health Sci. 2021;28(3):112–27.

[26] Arjvand F, Moeeni M, Najafi B, Nosratnejad S. Factors affecting inequality in the quality diets: a scoping review. Value Health Reg Issues. 2023;37:105–12. 37423079 10.1016/j.vhri.2023.05.003

[27] Mulatu S, Mulatu G, Gedif A. Dietary practice and associated factors among lactating mothers in Dangila District in the Awi Zone, Amhara region, Ethiopia, 2022: a cross-sectional study. Front Nutr. 2025;11:1–12. 10.3389/fnut.2024.1506707 PMC11743997 39834454

[28] Ministry of Public Health. Department of Health. Thai food-based dietary guidelines for preschool children. 2020.

[29] National Bureau of Agricultural Commodity and Food Standards (ACFS). Food consumption data of Thailand. Bangkok: Ministry of Agriculture and Cooperatives. 2016. https://www.m-society.go.th/ewtadmin/ewt/mso_web/article_attach/19305/20675.pdf. Accessed 14 Apr 2023.

[30] Wanisa Aorgart SP. The study of early childhood accordance with food behavioral consumption in case of the school in Bangkok. Srinakharinwirot Acad J Educ. 2016;17(2):13–27.

[31] Sroywong D, Wiboonwat J, Thongprachum A. Food consumption behavior among ethnic minority pre-school children in Mae Chaem District, Chiang Mai Province, Thailand. Thai J Public Health. 2022;52(2):103–12.

[32] Kusol K, Kaewpawong P, Eaksirinimit T, Hongsum T. Food consumption behaviors and growth of preschool children in child development centers, Thasala District, Nakhon Si Thammarat Province. J South Technol. 2023;16(1):41–53.

[33] World Health Organization. Physical status: the use and interpretation of anthropometry. Report of a WHO Expert Committee. Geneva: WHO. 1995. (Technical Report Series No. 854). 8594834

[34] World Health Organization. WHO Anthro (version 3.2.2, January 2011). Geneva: WHO; 2011.

[35] World Health Organization. Guideline: assessing and managing children at primary health-care facilities to prevent overweight and obesity in the context of the double burden of malnutrition: updates for the Integrated Management of Childhood Illness (IMCI). Geneva: World Health Organization; 2017. 29578661

[36] Suminar R, Fatimah S, Karim F. The culture of complementary feeding practice among stunting in toddlers aged under 24 months. Interdiscip Int J Conserve Cult. 2024;2(1):21–30.

[37] Mandara F, Festo C, Killel E, Lwambura S, Mrema J, Katunzi F, Martin HD, Elisaria E. The relationship between feeding practices and stunting among children under two years in Tanzania mainland: a mixed-method approach. Bull Natl Res Cent. 2024;48(1):112.

[38] WHO Nutrition and Food Safety Team. The double burden of malnutrition: policy brief. Geneva: World Health Organization; 2017.

[39] UNICEF, WFP, WHO. The state of food security and nutrition in the world 2025: addressing high food price inflation for food security and nutrition. Rome: FAO; 2025.

[40] Popkin BM. The nutrition transition and its health implications in lower-income countries. Public Health Nutr. 1998;1(1):5–21. 10555527 10.1079/phn19980004

[41] Mulyani AT, Khairinisa MA, Khatib A, Chaerunisaa AY. Understanding stunting: impact, causes, and strategy to accelerate stunting reduction—a narrative review. Nutrients. 2025;17(9):1493. 40362802 10.3390/nu17091493 PMC12073730

[42] Magno J, MC JGS, AdC, Pacheco LASME. Food frequency focuses on protein, carbohydrate, and minerals in children stunting aged 24–59 months at the Dom Aleixo Post Administrative, Dili Municipality, Timor-Leste, 2024. Asian J Health Sci. 2025;4(4):174–82.

[43] UNICEF. Improving young children’s diets during the complementary feeding period. In: UNICEF Programming Guidance. New York, NY: UNICEF. 2020.https://www.unicef.org/documents/improving-young-childrens-diets-during-complementary-feeding-period-unicef-programming. Accessed 23 June 2024.

[44] Inzaghi E, Pampanini V, Deodati A, Cianfarani S. The effects of nutrition on linear growth. Nutrients. 2022;14(9):1844. 35565716 10.3390/nu14091752 PMC9100533

[45] Mekonnen DA. Does household food and nutrient acquisition capacity predict linear growth in children? Analysis of longitudinal data from rural and small towns in Ethiopia. Food Secur. 2024;16(2):533–50.

[46] Jannati N, Mohammadi-Faez R, Mahmoodi MR, Azadbakht L. Association between quality and quantity of carbohydrate intake with selected anthropometric indices among primary school girls in Kerman city, Iran: a cross-sectional study. BMC Pediatr. 2024;24(1):1–10. 38658854 10.1186/s12887-024-04739-6 PMC11040773

[47] Elisanti AD, Jayanti RD, Amareta DI, Ardianto ET, Wikurendra EA. Macronutrient intake in stunted and nonstunted toddlers in Jember, Indonesia. J Public Health Res. 2023;12(3):1–9. 10.1177/22799036231197178 PMC10469258 37663312

[48] Ratnayani R, Sunardi D, Fadilah F, Hegar B. Nutrient intake and stunting in children aged 2–5 years in a slum area of Jakarta. Paediatr Indones. 2024;64(2):132–8.

[49] Mahfouz E, Mohammed E, Fadel S, Abd-el Rahman T. The relationship between dietary intake and stunting among preschool children in Upper Egypt. Public Health Nutr. 2021;25:1–23. 10.1017/S136898002100389X PMC9991819 34496999

[50] Basri H, Hadju V, Zulkifli A, Syam A, Ansariadi, Stang, Indriasari R, Helmiyanti S. Dietary diversity, dietary patterns and dietary intake are associated with stunted children in Jeneponto District, Indonesia. Gac Sanit. 2021;35:S483–6. 34929881 10.1016/j.gaceta.2021.10.077

[51] Oudat Q, Miller EL, Couch SC, Lee RC, Bakas T. Understanding caregivers’ influence on preschoolers’ eating behaviors: an integrative review guided by the theory of planned behavior. Child (Basel). 2025;12(2):163. 10.3390/children12020163 PMC11854435 40003266

[52] Watterworth JC, Hutchinson JM, Buchholz AC, Darlington G, Randall Simpson JA, Ma DWL, Haines J. Food parenting practices and their association with child nutrition risk status: comparing mothers and fathers. Appl Physiol Nutr Metab. 2017;42(6):667–71. 28196327 10.1139/apnm-2016-0572

[53] Srinivasan S, Dev PS, Eljo JOG. Intergenerational caregiving on grandparents’ health: a case study. SSRN Electron J. 2025.

[54] Jongenelis MI, Morley B, Worrall C, Talati Z. Grandparents’ perceptions of the barriers and strategies to providing their grandchildren with a healthy diet: a qualitative study. Appetite. 2021;159:105061. 33271201 10.1016/j.appet.2020.105061

[55] Schneiders ML, Phou M, Tum V, Kelley M, Parker M, Turner C. Grandparent caregiving in Cambodian skip-generation households: roles and impact on child nutrition. Matern Child Nutr. 2021;17(S1):e13127. 34241960 10.1111/mcn.13169 PMC8269139

[56] Tan C, Luo J, Zong R, Fu C, Zhang L, Mou J, Duan D. Nutrition knowledge, attitudes, behaviours and the influencing factors among nonparent caregivers of rural left-behind children under 7 years old in China. Public Health Nutr. 2010;13(10):1663–8. 20196906 10.1017/S1368980010000078

[57] Jongenelis MI, Budden T. The influence of grandparents on children’s dietary health: a narrative review. Curr Nutr Rep. 2023;12(3):395–406. 37329476 10.1007/s13668-023-00483-y PMC10444634

[58] Quak SL, Tong HJ, Hong CHL, Chong MFF, Duggal M, Amin Z, Gao X. Perspectives and influences of intergenerational caregivers on cariogenic feeding practices in childhood: a qualitative study. Int J Paediatr Dent. 2025;35(6):1055–70. 40338165 10.1111/ipd.13320 PMC12580897

[59] Zhang N, Bécares L, Chandola T, Callery P. Intergenerational differences in beliefs about healthy eating among carers of left-behind children in rural China: a qualitative study. Appetite. 2015;95:484–91. 26299714 10.1016/j.appet.2015.08.024

[60] Agustina K, Kesumawati K. The role of cultural values in feeding practices among children with stunting in Jembrana Regency: a qualitative approach. Babali Nurs Res. 2025;6:615–28.

[61] Jongenelis M, Morley B, Worrall C, Talati Z. Grandparents’ perceptions of the barriers and strategies to providing their grandchildren with a healthy diet: a qualitative study. Appetite. 2021;159:105061. 33271201 10.1016/j.appet.2020.105061

[62] Johnston D, Stevano S, Malapit HJ, Hull E, Kadiyala S. Time use as an explanation for the agri-nutrition disconnect: evidence from rural areas in low- and middle-income countries. Food Policy. 2018;76:8–18.

[63] Lioret S, Campbell KJ, McNaughton SA, Cameron AJ, Salmon J, Abbott G, Hesketh KD. Lifestyle patterns begin in early childhood, persist and are socioeconomically patterned, confirming the importance of early life interventions. Nutrients. 2020;12(3):1–15. 10.3390/nu12030724 PMC7146362 32182889

[64] Singer MR, Moore LL, Garrahie EJ, Ellison RC. The tracking of nutrient intake in young children: the Framingham Children’s Study. Am J Public Health. 1995;85(12):1673–7. 7503343 10.2105/ajph.85.12.1673 PMC1615722

[65] Rolland-Cachera MF, Deheeger M, Bellisle F, Sempé M, Guilloud-Bataille M, Patois E. Adiposity rebound in children: a simple indicator for predicting obesity. Am J Clin Nutr. 1984;39(1):129–35. 6691287 10.1093/ajcn/39.1.129

[66] Kato N, Ito T, Yokoya S, Tanaka T, Ono A, Yokomichi H, et al. Earlier BMI rebound and lower pre-rebound BMI as risks of obesity among Japanese preschool children. Int J Obes (Lond). 2018;42:52–8. 29064477 10.1038/ijo.2017.242

[67] Koyama S, Ichikawa G, Kojima M, Shimura N, Sairenchi T, Arisaka O. Adiposity rebound and the development of metabolic syndrome. Pediatrics. 2014;133(1):e114–9. 24366997 10.1542/peds.2013-0966

[68] Kepper M, Tseng T-S, Volaufova J, Scribner R, Nuss H, Sothern M. Pre-school obesity is inversely associated with vegetable intake, grocery stores and outdoor play. Pediatr Obes. 2016;11(5):e6–8. 26305391 10.1111/ijpo.12058 PMC4929036

[69] Newby PK. Plant foods and plant-based diets: protective against childhood obesity? Am J Clin Nutr. 2009;89(5):S1572–87. 10.3945/ajcn.2009.26736G 19321559

[70] Albani V, Butler LT, Traill WB, Kennedy OB. Fruit and vegetable intake: change with age across childhood and adolescence. Br J Nutr. 2017;117(5):759–65. 28367762 10.1017/S0007114517000599

[71] Demory-Luce D, Morales M, Nicklas T, Baranowski T, Zakeri I, Berenson G. Changes in food group consumption patterns from childhood to young adulthood: the Bogalusa Heart Study. J Am Diet Assoc. 2004;104(11):1684–91. 15499355 10.1016/j.jada.2004.07.026

[72] Magarey A, Mauch C, Mallan K, Perry R, Elovaris R, Meedeniya J, Byrne R, Daniels L. Child dietary and eating behavior outcomes up to 3.5 years after an early feeding intervention: the NOURISH RCT. Obes (Silver Spring). 2016;24(7):1537–45. 10.1002/oby.21498 27193736

[73] Wiriyasirikul S, Eksirinimit T, Kusol K, Jantasuwan R. The Effectiveness of Promoting a Vegetable and Fruit Consumption Behavior Program among Preschool Children in Nakhon Si Thammarat Province, Thailand. Sustainability. 2023;15(19):14350.

[74] Aekplakorn W, M-sL, Ruangdaraganon N, Satheannoppakao W, Puckcharern H. Thai Child National Health Examination Survey, NHES V. Nonthaburi: Health Systems Research Institute (HSRI); 2014.

[75] Worachina S, Thayansilp S, Khajornchaikul P. The influence of dietary behavior on multiple intelligences among disadvantaged preschool children aged 5–6 years in Surin Province. Res Dev J Loei Rajabhat Univ. 2018;13(45):44–55.

[76] Downs S, Demmler KM. Food environment interventions targeting children and adolescents: a scoping review. Glob Food Secur. 2020;27:100403.

[77] Onyeneke RU, Nwajiuba CA, Igberi CO, Umunna Amadi M, Anosike FC, Oko-Isu A, Munonye J, Uwadoka C, Adeolu AI. Impacts of caregivers’ nutrition knowledge and food market accessibility on preschool children’s dietary diversity in remote communities in Southeast Nigeria. Sustainability. 2019;11(6):1688.

[78] Fertig A, Glomm G, Tchernis R. The connection between maternal employment and childhood obesity: inspecting the mechanisms. Rev Econ Househ. 2009;7(3):227–55.

[79] Zozaya N, Oliva-Moreno J, Vallejo-Torres L. Association between maternal and paternal employment and their children’s weight status and unhealthy behaviours: does it matter who the working parent is? BMC Public Health. 2022;22:1331. 35821024 10.1186/s12889-022-13735-3 PMC9277834

