# Supplementary material for: Factors associated with a double burden of malnutrition among preschool-aged ethnic minority children in northern Thailand: a community-based cross-sectional study
Source: BMC Public Health. 2026 Feb 25;26:1082. doi: 10.1186/s12889-026-26777-8 (PMC13041485; doi:10.1186/s12889-026-26777-8)
Supplement: Supplementary file 1 — Supplementary Material 1. [file 12889_2026_26777_MOESM1_ESM.docx]

**Additional file**

**Table 1. Food consumption frequency categorized by food groups**

| **Food groups** | **Food items** | **Frequency of food consumption** | | | |
| --- | --- | --- | --- | --- | --- |
|  |  | **Never** | **1–2 times per week** | **3–4 times per week** | **Everyday** |
| **Grains and starches group** | Pizza, hamburger, French fries | 104(39.9) | 135(52.2) | 19(7.2) | 2(0.7) |
|  | Instant noodles (without vegetables or meat) | 33(12.7) | 143(55.0) | 66(25.4) | 18(6.9) |
|  | Rice noodles, egg noodles, “kuay jab” (rolled rice noodles soup) | 42(16.2) | 160(61.5) | 54(20.6) | 4(1.7) |
|  | Hainanese chicken rice, fried pork with rice, crispy pork with rice, roasted red pork with rice | 46(17.5) | 142(54.6) | 61(23.4) | 12(4.5) |
|  | Sticky rice with grilled chicken, fried chicken, grilled pork skewers | 38(14.8) | 123(47.4) | 83(32.0) | 15(5.8) |
|  | Rice porridge or congee (with pork, chicken, fish, shrimp, or pork liver) | 35(13.4) | 149(57.4) | 59(22.7) | 17(6.5) |
| **Vegetable group** | Battered and deep-fried vegetables, e.g., fried carrot, fried pumpkin | 139(53.6) | 86(33.0) | 28(10.7) | 7(2.7) |
|  | Boiled vegetables, e.g., vegetable clear soup | 37(14.1) | 80(30.9) | 90(34.7) | 53(20.3) |
|  | Stir-fried vegetables, e.g., ivy gourd, Chinese cabbage | 61(23.4) | 92(35.4) | 79(30.2) | 29(11.0) |
|  | Morning glory (water spinach) and mixed vegetables (equal portions) | 76(29.2) | 98(37.5) | 70(26.8) | 17(6.5) |
| **Fruit group** | Pickled fruits, e.g., mango, star gooseberry, tamarind | 179(68.7) | 63(24.1) | 14(5.5) | 4(1.7) |
|  | Canned fruits, e.g., rambutan, lychee, longan | 187(71.8) | 62(23.7) | 9(3.4) | 3(1.0) |
|  | Dried fruits, e.g., raisins, prunes | 187(71.8) | 62(23.7) | 11(4.1) | 1(0.3) |
|  | Fruit papaya salad, fruit salad | 147(56.4) | 84(32.3) | 24(9.3) | 5(2.1) |
|  | Very sweet fresh fruits, e.g., grapes, longan | 12(4.5) | 88(34.0) | 109(41.9) | 51(19.6) |
|  | Less sweet fruits, e.g., guava, papaya | 23(8.9) | 78(29.9) | 111(42.6) | 48(18.6) |
| **Meat, fish, and protein group** | Animal offal, e.g., pork liver | 23(8.9) | 78(29.9) | 111(42.6) | 48(18.6) |
|  | Fried or stir-fried meats with oil, e.g., fried pork, fried chicken, fried fish | 105(40.5) | 108(41.6) | 38(14.8) | 8(3.1) |
|  | Grilled or roasted meats, e.g., grilled chicken, grilled pork, grilled fish | 17(6.5) | 118(45.4) | 93(35.7) | 32(12.4) |
|  | Boiled or steamed meats, e.g., steamed fish, boiled pork, boiled squid | 34(13.1) | 143(55.0) | 72(27.8) | 11(4.1) |
|  | Processed meats, e.g., fried meatballs, imitation crab stick | 37(14.4) | 122(47.1) | 81(31.3) | 19(7.2) |
|  | Fried sausage | 14(5.2) | 109(41.9) | 95(36.4) | 43(16.5) |
|  | Boiled egg, steamed egg, eggs in brown soup (pa-lo), soft-boiled egg | 18(6.9) | 80(30.6) | 95(36.4) | 68(26.1) |
|  | Fried egg, omelet, grilled egg | 8(3.1) | 51(19.6) | 125(48.1) | 76(29.2) |
| **Milk and dairy products group** | Plain milk / Unsweetened milk | 12(4.8) | 38(14.8) | 48(18.6) | 161(61.9) |
|  | Flavored milk / Sweetened milk | 73(28.2) | 86(33.0) | 66(25.4) | 35(13.4) |
|  | Soy milk | 105(40.2) | 88(34.0) | 47(18.2) | 20(7.6) |
|  | Yogurt | 72(27.8) | 128(49.1) | 47(17.9) | 14(5.2) |
|  | Fermented milk / Cultured milk drink | 52(19.9) | 107(41.2) | 72(27.8) | 29(11.1) |
| **Snacks and beverages group** | Bakery products, e.g., cake, pie, donut, Japanese crepe, sandwich | 47(17.9) | 160(61.5) | 39(15.1) | 14(5.5) |
|  | Sweet snacks, e.g., ice cream, chocolate, candy, jelly, chewing gum | 14(5.2) | 99(38.1) | 88(33.7) | 60(23.0) |
|  | Sweetened beverages, e.g., soft drinks, sweet syrup drinks, iced cocoa, fruit smoothies | 27(10.3) | 113(43.3) | 81(31.3) | 39(15.1) |
|  | Savory snacks, e.g., seasoned dried fish strips | 6(2.4) | 82(31.6) | 77(29.6) | 95(36.4) |
